# Machine Learning-Based Predictive Models for Detection of Cardiovascular Diseases

**DOI:** 10.3390/diagnostics14020144

**Published:** 2024-01-08

**Authors:** Adedayo Ogunpola, Faisal Saeed, Shadi Basurra, Abdullah M. Albarrak, Sultan Noman Qasem

**Affiliations:** 1 DAAI Research Group, College of Computing and Digital Technology, Birmingham City University, Birmingham B4 7XG, UK; adedayo.ogunpola@mail.bcu.ac.uk (A.O.); shadi.basurra@bcu.ac.uk (S.B.); 2 Computer Science Department, College of Computer and Information Sciences, Imam Mohammad Ibn Saud Islamic University (IMSIU), Riyadh 11432, Saudi Arabia; amsbarrak@imamu.edu.sa (A.M.A.); snmohammed@imamu.edu.sa (S.N.Q.)

**Keywords:** cardiovascular diseases, deep learning, disease detection, heart diseases, machine learning, ensemble learning, XGBoost

## Abstract

Cardiovascular diseases present a significant global health challenge that emphasizes the critical need for developing accurate and more effective detection methods. Several studies have contributed valuable insights in this field, but it is still necessary to advance the predictive models and address the gaps in the existing detection approaches. For instance, some of the previous studies have not considered the challenge of imbalanced datasets, which can lead to biased predictions, especially when the datasets include minority classes. This study’s primary focus is the early detection of heart diseases, particularly myocardial infarction, using machine learning techniques. It tackles the challenge of imbalanced datasets by conducting a comprehensive literature review to identify effective strategies. Seven machine learning and deep learning classifiers, including K-Nearest Neighbors, Support Vector Machine, Logistic Regression, Convolutional Neural Network, Gradient Boost, XGBoost, and Random Forest, were deployed to enhance the accuracy of heart disease predictions. The research explores different classifiers and their performance, providing valuable insights for developing robust prediction models for myocardial infarction. The study’s outcomes emphasize the effectiveness of meticulously fine-tuning an XGBoost model for cardiovascular diseases. This optimization yields remarkable results: 98.50% accuracy, 99.14% precision, 98.29% recall, and a 98.71% F1 score. Such optimization significantly enhances the model’s diagnostic accuracy for heart disease.

## 1. Introduction

The heart plays a crucial role in sustaining life by effectively pumping oxygenated blood and regulating important hormones to maintain optimal blood pressure levels. Any deviation from its functioning can lead to the development of heart conditions, collectively known as cardiovascular diseases (CVD). CVD includes a range of disorders that affect both the heart and blood vessels, such as cerebrovascular problems, congenital anomalies, pulmonary embolisms, irregular heart rhythms (arrhythmias), peripheral arterial issues, coronary artery disease (CAD), rheumatic heart ailments, coronary heart disease (CHD), and cardiomyopathies that affect the heart muscle. 

Notably, CHD is the subtype among cardiovascular diseases, accounting for a significant 64% of all cases. While it primarily affects men, women are also susceptible to its impact. Within the realm of CVDs, CAD is particularly concerning due to its association with global mortality rates. According to the World Health Organization (WHO) [1], the consequences of CVDs are profound, with staggering statistics indicating an estimated 17.9 million deaths annually are attributed to these diseases worldwide. These alarming numbers highlight the significance of research efforts and medical advancements dedicated to combatting and lessening the impact of cardiovascular diseases worldwide. There are risk factors that contribute to the development of CVDs, including blood pressure, excess body weight and obesity, abnormal lipid profiles, glucose irregularities or diabetes conditions, tobacco usage or smoking habits, physical inactivity or sedentary lifestyle, alcohol consumption, and cholesterol levels. The WHO predicts that CVD will remain a cause of mortality, silently posing a substantial threat to human life for the foreseeable future, possibly even beyond 2030. 

Machine learning, as highlighted by Ramesh et al. [2], enjoys major transformative capability within the healthcare industry. Its outstanding advancements can be ascribed to its exceptional data processing abilities, which are far superior to those of humans. Consequently, the field of healthcare has observed the development of several AI applications that leverage machine learning’s speed and accuracy, paving the way for revolutionary solutions to diverse healthcare challenges. Several machine learning methods have been applied for the purpose of detecting cardiovascular diseases. However, there is still a need to enhance the predictive models and address the research gaps in the existing detection approaches, such as the challenge of imbalanced datasets, which can lead to biased predictions. 

By investigating the effectiveness of hybrid models combining different techniques, various researchers have explored diverse methodologies, including neural networks and various machine learning methods, to enhance prediction accuracy [3,4,5,6,7,8,9,10,11,12]. While these studies provide valuable insights, the variability in datasets, models, and outcomes underscores the complexity of the predictive task. Despite the advancements, there remains a pressing need for further investigations to refine existing models and improve the overall performance of cardiovascular disease prediction. The diverse landscape of machine learning applications in this domain emphasizes the importance of continued research to enhance the accuracy, reliability, and generalizability of predictive models, ultimately contributing to more effective clinical interventions and patient care.

In this paper, we have explored the strengths and limitations of the existing machine learning (ML) techniques in the context of heart disease analysis. Then, we investigated and applied seven machine learning-driven predictive models that can enhance the detection of cardiovascular and cerebrovascular diseases; these models include K-Nearest Neighbors, Support Vector Machine, Logistic Regression, Convolutional Neural Network, Gradient Boost, XGBoost, and Random Forest. Two datasets were used in this study, which were pre-processed using different techniques such as oversampling, feature scaling, normalization, and dimensionality reduction to optimize data for effective machine learning analysis. Finally, we evaluated and compared the efficacy of different machine learning (ML) techniques for analyzing heart diseases within the healthcare sector.

## 2. Related Works

In this paper, we present a concise technical background and review pertinent literature related to research studies conducted on the early forecast of heart disease utilizing machine learning and deep learning techniques. We highlight the different methods that have been employed in these studies to foretell heart disease at an initial stage. 

### 2.1. Machine Learning Approach

Machine learning remains a rapidly advancing discipline of computational algorithms that try to imitate human intelligence by learning through data and the surrounding environment. These algorithms play a crucial role in processing and analyzing large-scale data, often referred to as “big data”. Machine learning techniques have demonstrated their effectiveness in various domains, including pattern recognition, computer vision, spacecraft engineering, as well as biomedical and medical applications. Their versatility and success have made them indispensable tools in addressing complex challenges and extracting valuable insights from diverse datasets [13]. 

Machine learning is a specialized approach that automates the process of model building. Using algorithms, machines can discover hidden patterns and insights within datasets. Importantly, in machine learning, we do not particularly instruct machines on where to explore for insights; instead, the algorithms enable the machines to learn and adapt their techniques and outputs as they uncover new-found data and scenarios. This iterative nature of machine learning allows for continuous improvement and adaptation, making it a powerful tool for processing and analyzing complex datasets [14]. 

There exist two main approaches in machine learning: supervised learning and unsupervised learning. In one approach, supervised learning, algorithms are trained using specific examples. The machine is provided with input data along with their corresponding correct outputs. Learning takes place by comparing the machine’s experimental outcomes with the accurate outputs to discover blunders. This sort of learning is suitable after previous data has been utilized to foretell future occurrences [15]. 

The other approach, unsupervised learning, involves the machine exploring the records and attempting to discover patterns or structures on its own. It needs to create models commencing from scratch and is not provided with any precise outputs to guide its learning process. Unsupervised learning is commonly employed to detect and distinguish outliers in the data. This approach is particularly useful when there is limited or no labeled data available for training [14]. Researchers worldwide have made significant efforts to combat cardiovascular disease (CVD) and improve patient outcomes [16]. These efforts include enhancing clinical decision support systems to achieve precise early detection and enable effective treatment. Machine learning (ML) and artificial intelligence (AI) techniques have played a pivotal role in the early detection and diagnosis of CVD. 

CVD detection encompasses different distinct approaches. The first approach involves utilizing AI models that analyze various test reports to distinguish between CVD patients and healthy citizens. The second approach utilizes signals such as electrocardiogram (ECG) and heart sound signals as vital information for ML models to classify individuals as either healthy or having CVD [16]. 

### 2.2. Deep Learning Approach 

In recent years, there has been remarkable progress in the field of deep learning, with a primary focus on developing intelligent automated systems that aid doctors in predicting and diagnosing diseases through the utilization of the Internet of Things (IoT). While conventional machine learning techniques were often restricted by their dependency on single datasets, the advent of deep learning has brought significant enhancements to the accuracy of existing algorithms. Deep learning leverages artificial neural networks, which consist of multiple hidden layers organized in a cascading pattern. This architecture enables the processing of non-linear datasets, allowing for more complex patterns and relationships to be captured and learned by the model. As a result, deep learning has emerged as a powerful tool in medical applications, providing improved predictive capabilities and enhancing disease diagnosis through the integration of IoT devices and data sources. This approach has shown promising results, outperforming older machine learning algorithms in terms of accuracy. As accurate medical support systems for detecting hidden patterns and predicting diseases are still lacking, deep learning offers the potential to accurately predict heart diseases at an early stage, allowing for timely intervention and treatment [17]. 

Sudha and Kumar [18] observed that the Convolutional Neural Network (CNN) is a suitable method for diagnosing heart disease. CNN’s ability to learn and represent features in a concise and conceptual manner is advantageous, especially as the network’s depth increases. Additionally, they proposed a hybrid model that combines Convolutional Neural Networks (CNN) with Long Short-Term Memory (LSTM) units, which are a type of recurrent neural network (RNN). LSTM units are known for their ability to store and transmit relevant information over long sequences, making them particularly useful for time-series data such as heart disease data. By integrating CNN and LSTM, the hybrid model aimed to enhance the accuracy of heart disease classification. The CNN component is adept at capturing spatial patterns in the data, while the LSTM component excels at recognizing temporal dependencies and patterns. This combination allows the model to effectively learn complex features from the data, leading to improved classification accuracy. Experimental results from the study revealed promising outcomes, with the hybrid model achieving an accuracy of 89%, sensitivity of 81%, and specificity of 93%. These results outperformed conventional machine learning classifiers, indicating the potential of the proposed hybrid approach in advancing the accuracy of heart disease classification [18]. 

The healthcare sector has emerged as a prime beneficiary of the growing volume and accessibility of data [19]. Various entities, such as healthcare providers, pharmacological firms, research institutions, and government parastatals, are now accumulating vast volumes of data from diverse sources, including research, clinical trials, public health programs, and insurance data. The merging of such data holds immense potential for advancing healthcare practices and decision-making [20]. Traditionally, doctors used to diagnose and treat patients based on their symptoms alone. However, evidence-based medicine has become the prevailing approach, where physicians review extensive datasets obtained from medical trials and treatment paths on a huge scale to make decisions built on the most comprehensive and up-to-date information available. This shift towards data-driven decision-making is transforming healthcare practices, improving patient outcomes, and driving further advancements in the medical field [14]. 

Numerous industry and research initiatives are actively working on implementing machine learning expertise in the healthcare sector to enhance patient care and well-being globally. One such initiative is the Shah Lab, based at Stanford University [14]. The Shah Lab focuses on leveraging machine learning and data science to address critical healthcare challenges and develop innovative solutions for various medical applications. Through these initiatives, researchers and experts aim to harness the power of machine learning to analyze large-scale healthcare data, including electronic health records, medical imaging, genomics, and patient outcomes. By extracting valuable insights and patterns from this data, they aim to improve disease diagnosis, treatment prediction, personalized medicine, and overall patient management. The goal is to provide healthcare professionals with advanced tools and technologies that can assist them in making more accurate and timely clinical decisions, leading to better patient outcomes and an overall improvement in healthcare services worldwide. Table 1 below presents a summary of the performance metrics related to the existing methods under evaluation, with each entry corresponding to specific evaluation criteria.

### 2.3. Datasets Collection and Preprocessing 

In their study, Algarni et al. [27] utilized a dataset of coronary artery X-ray angiography images obtained from a clinical database. These images exhibited challenging characteristics, including uneven vessel thickness, complex vascular structures in the background, and the presence of noise. The dataset consisted of 130 X-ray coronary angiograms, each having a size of 300 × 300 pixels. The data was collected from the cardiology department of the Mexican Social Security Institute, and ethical approval was obtained (reference number R-2019-1001-078) for the use of this medical database in heart disease diagnosis. To train and evaluate their proposed model, called ASCARIS, the dataset was randomly divided into two parts: a training set containing 100 images and a test set comprising 30 images. The ASCARIS model was developed based on color, diameter, and shape features extracted from the angiography images. 

Al Mehedi et al. [28] utilized a dataset of 299 heart failure patients obtained from the Faisalabad Institute of Cardiology and the Allied Hospital in Faisalabad. The dataset consisted of 13 attributes, including features such as Age, Anemia, High Blood Pressure, Creatinine Phosphokinase (CPK), Diabetes, Ejection Fraction, Sex, Serum Creatinine, Serum Sodium, Smoking, Time, and a target column labeled as “Death Event”, which was used for binary classification. The dataset underwent preprocessing to ensure its quality and consistency. After preprocessing, the dataset was divided into separate train and test sets for model training and evaluation. Two feature selection methods were applied to the train set to identify the most relevant features for the heart failure prediction task. 

Deepika and Seema [29] conducted a study on heart disease with datasets available online from the UCI Machine Learning Repository at the University of California, Irvine. They comprise 76 attributes, including the target property, but only 14 of these attributes were considered essential for analysis. The researchers used two specific datasets for their study: the Cleveland Clinic Foundation dataset, with records from 303 patients, and the Hungarian Institute of Cardiology dataset, with records from 294 patients. Various machine learning algorithms, including Naïve Bayes (NB), Support Vector Machine (SVM), Decision Tree (DT), and Artificial Neural Networks, were employed in the analysis to predict heart disease. Within the broader context, Table 2 clarifies the preprocessing approaches and predictive methodologies utilized in previous studies.

### 2.4. Discussions on the Research Limitations 

The literature review involved an in-depth exploration of the existing research and knowledge pertaining to heart disease prediction using diverse machine learning and deep learning techniques. Several studies reviewed the recent advancements and limitations of applying machine learning for cardiovascular disease detection [10,33,34,35,36]. For instance, the studies [8,37,38,39,40] proposed different data mining and machine learning methods based on heartbeat segmentation and selection process, ECG images, images of carotid arteries, and others. 

Numerous studies have concentrated on applying machine learning algorithms such as Decision Tree, Naïve Bayes, Random Forest, Support Vector Machine, and Logistic Regression on the Heart Disease Dataset, yielding promising accuracy rates for classification. Moreover, deep learning methods, particularly Convolutional Neural Networks (CNN), have gained significant traction for effectively handling complex tasks and unstructured data. The review also examined discussions regarding the implementation of data preprocessing techniques, feature selection methods, and performance evaluation metrics to optimize the efficiency of predictive models. Some studies underscored the importance of data quality and the relevance of specific features in enhancing the accuracy of the models. 

Machine learning algorithms play a crucial role in precisely foretelling heart disease by discovering suppressed patterns in data, making predictions, and improving performance based on historical data. These programs make it possible for us to anticipate and diagnose heart disease more accurately, while deep learning, fueled by artificial neural networks, is a critical factor in handling complex computations on large volumes of data. These algorithms play an essential role in identifying key attributes and patterns in both structured and unstructured data, enhancing more efficient data analysis and processing. 

Employing machine learning and deep learning approaches offers considerable potential in the field of heart disease diagnosis and treatment. These sophisticated techniques enable the integration of various data sources, such as medical records, imaging data, genetics, and lifestyle factors, to create a universal and individualized approach to healthcare. The iterative nature of machine learning acknowledges continuous learning and adaptation, resulting in progressed diagnostic and predictive models over time. This promises to enhance the accuracy and effectiveness of heart disease management, ultimately leading to better patient outcomes. 

After reviewing the available literature, it is evident that there is a lack of extensive experimentation on the use of Gradient Boosting models in the detection of heart disease. However, considering the unique capabilities of Gradient Boosting models in analyzing data and capturing temporal dependencies, their potential in this domain is worth exploring. 

The potential of Gradient Boosting models to progressively enhance predictive accuracy by refining weaker learners within the model positions them as promising contenders for improving the precision of heart disease detection. Consequently, there is a need for further exploration and experimentation dedicated to harnessing the capabilities of Gradient Boosting models in this context. 

By embracing the use of Gradient Boosting models in heart disease detection and conducting more targeted experiments, we can unlock new possibilities for advancing healthcare interventions and ultimately enhancing patient outcomes and well-being. 

## 3. Materials and Methods

**Figure 1:**
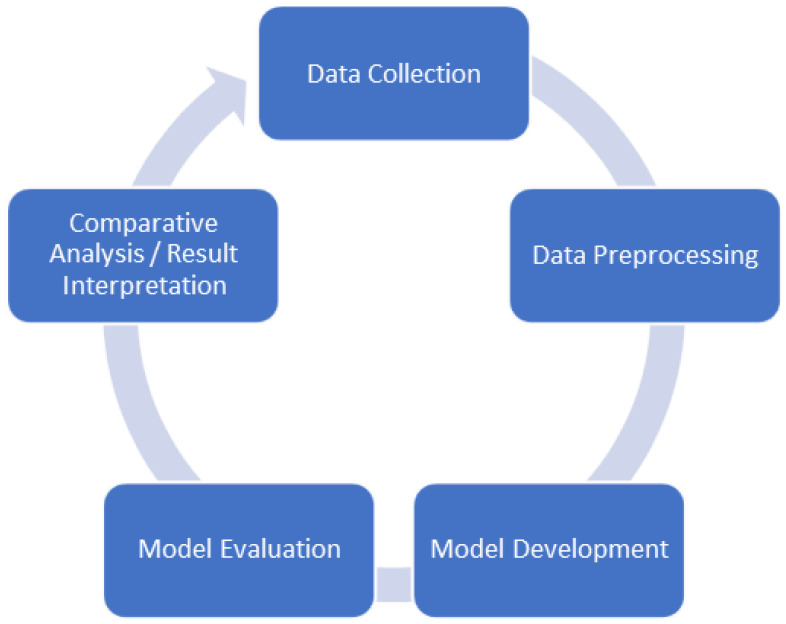
Research method workflow.

The following methods are adapted to achieve the goals of this research. They are applied to explore and comprehend various dimensions of heart-related conditions, ultimately contributing to the creation of precise models for the diagnosis and prediction of these conditions. The general research method framework of this study is shown in Figure 1. 

### 3.1. Datasets

To carry out this research study, two datasets were examined, namely the Cardiovascular Heart Disease Dataset, which was retrieved from the Mendeley database, and the Heart Disease Cleveland Dataset, which was retrieved from the Kaggle database. The “Cardio” and “Target” columns on both datasets refer to the column we are trying to predict with numeric values 0 (no disease) and 1 (disease). It is important to note that neither dataset has any missing values. The detailed descriptions of all these attributes are listed below: 

The Cardiovascular Heart Disease Dataset (Table 3) holds significant importance within the healthcare and machine learning domains. It serves as an asset for tasks associated with the prediction and classification of cardiovascular diseases while holding data of 1000 data samples in 13 attributes, each representing a potential risk factor. 

Shifting our focus to the Heart Disease Cleveland Dataset (Table 4), a widely recognized dataset frequently employed in the fields of machine learning and healthcare, which has been extensively used in tasks related to predicting and classifying heart disease. This dataset holds prominence for its pivotal role in assessing the effectiveness of diverse machine learning algorithms in diagnosing heart disease with 303 patients’ information in 14 attributes. Its primary objective revolves around predicting whether heart disease is present or absent. 

### 3.2. Data Pre-Processing

Data preprocessing is an essential step within machine learning that aims to improve dataset quality and reliability before analysis and modeling. This phase tackles challenges such as missing data, inconsistencies, outliers, and skewed class distributions. Addressing missing values is crucial to ensure accurate insights by utilizing techniques such as imputation. Detecting and managing outliers is also vital, as these data points can skew results. A key concern is class distribution balance, where methods like oversampling mitigate imbalanced datasets. Considering these considerations, employing techniques such as feature scaling, normalization, and dimensionality reduction can optimize data for effective machine learning analysis. 

### 3.3. Model Development 

The conclusion of the thorough literature work brings us to the pivotal stage of model development. This section encompasses seven notable machine learning techniques: Logistic Regression, Convolutional Neural Network, Support Vector Machine (SVM), Gradient Boosting, K-Nearest Neighbors (KNN), XGBoost, and Random Forest. Each algorithm contributes distinct characteristics to unveil predictive revelations in the analysis of cardiovascular and cerebrovascular diseases, utilizing resources such as Scikit-Learn and Keras libraries. 

Each of these models possesses unique traits, spanning from linear approaches to ensemble techniques and deep learning architectures. Through thorough empirical investigations, we assessed the effectiveness of every model in terms of recall, precision, accuracy, and F1-score metrics. 

### 3.4. Model Evaluation 

Model Evaluation stands as a pivotal phase in the realm of machine learning, dedicated to thoroughly gauging how well-trained models predict outcomes. This essential step ensures that models can generalize to new data effectively, informing decisions about deployment and refinement. The following key techniques and metrics will contribute to a comprehensive evaluation of this study: 

Confusion Matrix: Offering insight into true positives, true negatives, false positives, and false negatives, this matrix forms the basis for calculating vital metrics. 

Accuracy: Providing an overall view of model performance by measuring correctly predicted instances against the total dataset.

Accuracy = (TP + TN)/(TP + FP + TN + FN)(1)

Precision and Recall: Precision assesses positive prediction accuracy, while recall gauges the model’s ability to capture positive instances.

Precision = TP/(TP + FP)(2)

Recall = TP/(TP + FN)(3)

F1-Score: Striking a balance between precision and recall, this score is essential for harmonizing performance aspects.

F1 = (2 × precision × recall)/(precision + recall)(4)

Cross-Validation: This technique partitions data for training and testing, guarding against overfitting. 

Hyperparameter Tuning: Optimizing model parameters through techniques like GridSearch enhances performance. 

## 4. Results

This section explores the detailed analysis of machine learning models for heart disease prediction, leveraging two distinct datasets: the Cardiovascular Heart Disease Dataset and the Heart Disease Cleveland Dataset using the Python programming language. 

Our primary objective is to identify the most effective predictive models, considering both traditional tabular datasets while keeping in mind the aims of the study. 

### 4.1. Pre-Processing Results 

To harness the potential of the Cardiovascular Heart Disease Dataset and the Heart Disease Cleveland Dataset for machine learning applications, it becomes imperative to execute preliminary data preprocessing procedures. These procedures encompass a range of actions, including managing missing data, encoding categorical variables, standardizing or normalizing feature values, and partitioning the dataset into distinct training and testing subsets. Additionally, the utilization of exploratory data analysis (EDA) techniques and data visualization tools proves instrumental in gaining insights into data distributions and inter-variable relationships. 

**Figure 2:**
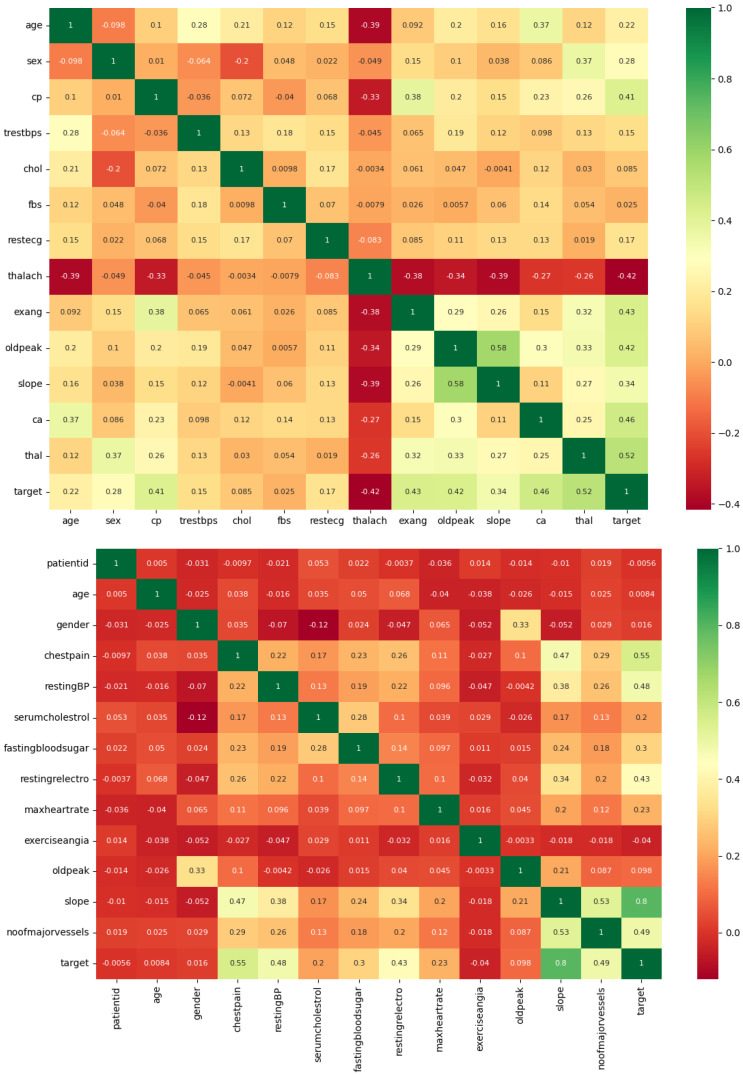
Heatmap distribution of the dataset features.

Firstly, a correlation matrix heatmap is created, as shown in Figure 2. This heatmap computes the correlation coefficients among diverse attributes in the datasets and represents them graphically. Its purpose is to facilitate the visual examination of associations between various features. Positive correlations are depicted using green hues, whereas negative correlations are represented in red. This heatmap serves the purpose of identifying the features that exhibit the most substantial correlations with the target variable, thereby revealing their impact on the presence or absence of cardiovascular disease. On the left side is the Cardiovascular Heart Disease Dataset, while on the right is the Heart Disease Cleveland Dataset. 

**Figure 3:**
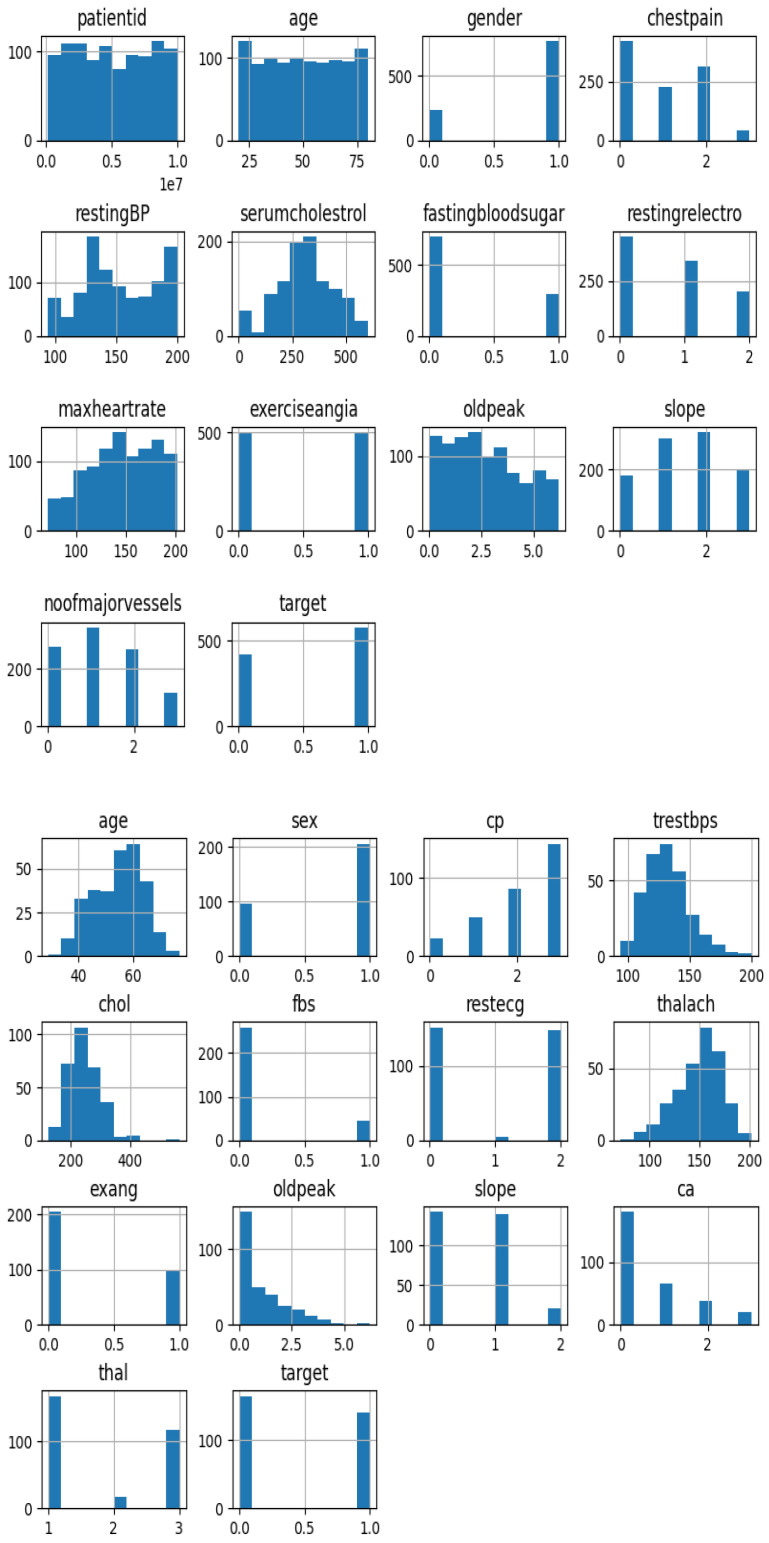
Histogram distribution of the dataset features.

The histograms corresponding to individual dataset attributes provide valuable insights by allowing exploration of each feature’s distribution, as shown in Figure 3. They are instrumental in the detection of potential outliers and provide a rapid overview of the characteristics and spans of these features. This visualization is a helpful tool for comprehending the overall shape and distribution of the data. The pictorial evidence of both datasets can be seen below, where the Cardiovascular Heart Disease Dataset is on the left, and the Heart Disease Cleveland Dataset can be seen on the right. 

**Figure 4:**
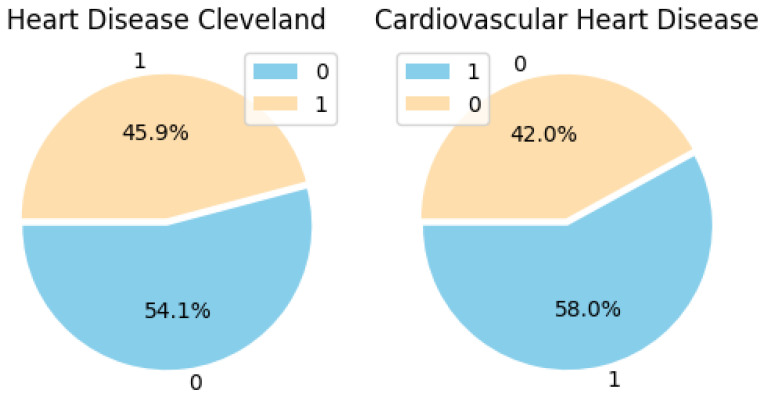
The distribution of features in the target variable.

As shown in Figure 4, the pie chart is utilized to depict the distribution of the target variable, which signifies the existence or non-existence of cardiovascular disease. The figure shows the distribution of features in the target variable, where 1 represents features with heart disease, and 0 represents features without heart disease. It enumerates the instances of each class and exhibits the proportions as percentages in the pie chart, illustrating the presence and absence of cardiovascular disease. In Figure 4, the pie chart on the right represents features of the target column distribution of the Cardiovascular Heart Disease Dataset, while the left represents the feature of the target column distribution of the Heart Disease Cleveland Dataset. 

After successfully preprocessing and visualizing the features of the dataset, we conducted an in-depth exploration of various machine learning models to discern their predictive efficacy. 

### 4.2. K-Nearest Neighbors (KNN) Results 

We commenced the analysis by employing the K-Nearest Neighbors (KNN) algorithm with varying ‘k’ values, representing the number of nearest neighbors considered during the predictions. Employing cross-validation, we computed scores for each ‘k’ value, ultimately discerning that ‘k = 7’ yielded the most favorable mean cross-validation score. This outcome underscores that configuring KNN with ‘k = 7’ exhibits significant promise. 

As shown in Table 5, Table 6, Table 7 and Table 8, the implementation of this model yielded an impressive accuracy rate of 96.50% and 91.80% on the datasets, respectively, serving as an overarching measure of the model’s correctness in its predictions. Furthermore, meticulous hyperparameter tuning was carried out to guarantee optimal performance. The precision score, gauging the proportion of true positive predictions among all positive predictions, achieved a notable level, approximately 96.61% and 96.55%. Additionally, the recall, representing the proportion of true positive predictions among all actual positives, exhibited a strong value, approximately 97.44%, and 87.50%. Similarly, the F1 Score attained an impressive value, hovering around 97.02% and 91.80%. These metrics collectively affirm the exceptional performance of the KNN model within the dataset. 

### 4.3. Random Forest Results 

By conducting an extensive hyperparameter tuning process, we modified the number of trees (n_estimators) to 200 within the Random Forest ensemble model. As shown in Table 5, Table 6, Table 7 and Table 8, the tuned model achieved an outstanding accuracy level, hovering at around 98.60% and 91.09%. The assessment of precision showed a significant enhancement, which obtained 98.63% and 94.44%.

Similarly, the F1 Score, which amalgamates precision and recall, demonstrated the model’s robustness, registering a value of 98.80% and 89.81, respectively. Furthermore, the recall score, measuring the model’s aptitude for recognizing genuine positive cases, reached a remarkable value of 98.97% and 85.61. 

### 4.4. Logistic Regression (LR) Results 

By implementing a custom threshold of 0.6, the model was configured to adopt a cautious approach when classifying instances as positive. To be specific, if the predicted probability of an instance belonging to the positive class (class 1) equaled or exceeded 0.6, it was categorized as positive; otherwise, it was designated as negative. This threshold selection significantly influenced how the model struck a balance between precision and recall. As shown in Table 5, Table 6, Table 7 and Table 8, the model’s precision score was 96.55% and 93.10%, signifying its proficiency in minimizing false positive predictions. 

The recall scores stood at 95.73% and 84.38%, emphasizing the model’s importance in correctly identifying all positive cases, particularly in scenarios where missing potential cases of heart disease is a critical concern. The F1 Score captured genuine positive cases at 96.14% and 88.52%. Regarding overall accuracy, the model achieved an accuracy score of 95.50% and 88.52%. 

### 4.5. Gradient Boosting (GB) Results 

Through the GridSearchCV process, we effectively fine-tuned the model’s hyperparameters. The optimal hyperparameters selected encompassed a learning rate of 0.2, a maximum depth of 3 for individual trees, and 100 boosting stages (n_estimators). These hyperparameters were chosen based on their exceptional performance on the validation datasets. When tested on independent data, the refined Gradient Boosting model consistently delivered exceptional results. As shown in Table 5, Table 6, Table 7 and Table 8, it attained an impressive precision score of 99.13% and 90.90%, indicative of its ability to minimize false positive predictions effectively. 

Furthermore, the model exhibited a recall score of 97.44% and 84.38%, which holds paramount importance in medical applications where identifying potential cases of heart disease is critical. The F1 Score, which harmonizes precision and recall, reached an impressive value of 98.28% and 87.10. 

The model’s accuracy on the test dataset was consistently high, measuring 98.00%, although it achieved 86.89% on the Heart Disease Cleveland Dataset. These findings collectively underscore the Gradient Boosting model’s exceptional suitability for the task of heart disease classification, highlighting its potential to accurately detect individuals with heart disease while maintaining a low rate of false positives. Such performance makes it an asset for healthcare professionals and researchers in the cardiology field. 

### 4.6. Support Vector Machine (SVM) Results 

The process of tuning hyperparameters, carried out through GridSearchCV, effectively determined the most suitable hyperparameter configuration for the SVM model. This configuration included a regularization parameter (C) set to 10, a polynomial kernel with a degree of 2, and the utilization of a linear kernel. 

As shown in Table 5, Table 6, Table 7 and Table 8, for post-tuning, the model achieved a precision score of 95.00% and 80.65%, a recall score of 97.44% and 78.12%, and an F1 Score of 96.20% and 79.37%. 

On the test dataset, the model exhibited an accuracy of approximately 95.50% and 78.69%, affirming its consistent and accurate predictive capabilities. 

### 4.7. Convolutional Neural Network (CNN) Results

The model architecture consists of three layers: an initial layer with 128 units employing the ReLU activation function, followed by a hidden layer featuring 64 units with ReLU activation, and ultimately, an output layer utilizing the sigmoid activation function. During model compilation, the Adam optimizer was employed alongside binary cross-entropy loss, with accuracy serving as the evaluation metric. 

To mitigate the risk of overfitting, a precautionary measure known as early stopping was integrated into the training process. This involved monitoring the validation loss for a maximum of 10 epochs and restoring the model’s weights to their best configuration. The training was conducted using scaled training data over a maximum of 100 epochs, employing a batch size of 64. 

As shown in Table 5, Table 6, Table 7 and Table 8, the model’s performance on the test dataset is particularly noteworthy. Precision achieved an impressive score of 97.46% and 87.50%. 

This suggests that when the model predicts an individual as having heart disease, it is highly likely to be accurate. Furthermore, the recall scores were 98.29% and 87.50%. The F1 Score demonstrates resilience at 97.87% and 87.50%. Overall accuracy, which reflects the ratio of correctly predicted cases to the total cases, stands at 97.50% and 86.89%, respectively. 

### 4.8. XGBoost Results 

Through the utilization of GridSearchCV, a highly effective process of hyperparameter tuning was carried out. This process led to the discovery of optimal hyperparameters for the XGBoost model, which included a learning rate of 0.2, a maximum tree depth of 3, 100 boosting rounds (n_estimators), and a subsample fraction of 1.0. The recall of these chosen hyperparameters was substantiated by a remarkable validation score of approximately 98.00% on the Cardiovascular Heart Disease Dataset and 84% on the Heart Disease Cleveland Dataset, respectively. 

On the test dataset, the fine-tuned XGBoost model upheld its exceptional performance by achieving a precision score of 99.14% and 90.00%, signifying its adeptness in accurately categorizing positive cases. Moreover, the recall score, at 98.29% and 84.38%, holds particular significance. The F1 Score exhibits resilience at 98.71% and 87.10%. The model’s overall accuracy on the test data hovers at 98.50% and 86.89%. These remarkable outcomes underscore the XGBoost model’s aptness for heart disease classification. 

## 5. Discussion 

**Figure 5:**
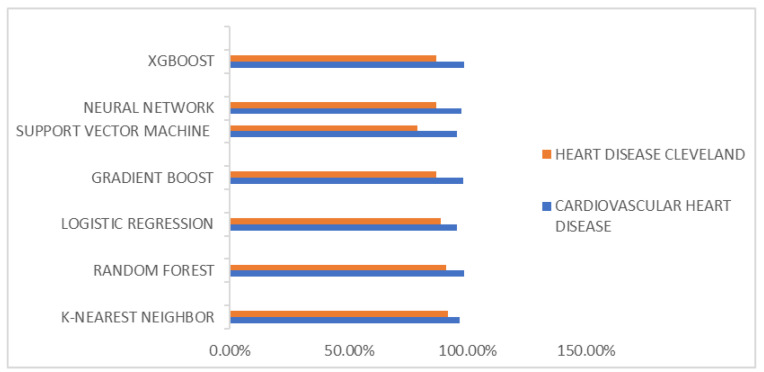
Accuracy of machine learning models on both datasets.

The experimental results are shown in Table 5, Table 6, Table 7 and Table 8 and Figure 5. The thorough assessment of machine learning models, specifically the XGBoost and K-Nearest Neighbors models, in the context of heart disease prediction, provides valuable insights. These insights align with the research conducted by Zhang et al. [41], which underscores the effectiveness of the XGBoost algorithm in this specific domain. 

Across both datasets, these models consistently demonstrate exceptional performance, emphasizing their efficacy in heart disease prediction. Notably, the XGBoost model stands out with an impressive accuracy rate of 98.50% in the Cardiovascular Heart Disease Dataset, while the K-Nearest Neighbors (KNN) model achieves a commendable accuracy of 91.80% in the Heart Disease Cleveland Dataset. These high levels of accuracy emphasize the models’ reliability, positioning them as valuable tools for diagnosing heart disease. 

Precision, a critical metric in healthcare, reflects the models’ ability to identify heart disease cases precisely. Both models achieve outstanding precision, with the XGBoost model leading at 99.14%, closely followed by the KNN model at 96.55%. These elevated precision levels significantly reduce the occurrence of false positive diagnoses, alleviating unnecessary concerns for patients. 

Furthermore, the F1 Score, which balances precision and recall, highlights the XGBoost model’s effectiveness in recognizing heart disease cases while minimizing the risk of overlooking positive instances. The model achieves F1 Scores of 98.71% and 91.80% in both datasets, showcasing its ability to strike this delicate balance effectively. 

## 6. Conclusions and Future Scope

As we discussed the broader scope of model selection and its implications for heart disease prediction, the conducted analysis has unearthed invaluable insights. Among the array of models under scrutiny, K-Nearest Neighbors and XGBoost have consistently risen to prominence as top-performing candidates across both datasets, as shown below. These models have exhibited remarkable accuracy and recall scores, rendering them robust contenders for the precise classification of heart disease. It is noteworthy, however, that other models, including Logistic Regression, Convolutional Neural Network, Gradient Boost, Random Forest (RF), and Support Vector Machines (SVM), have showcased significant predictive capabilities once their hyperparameters were meticulously tuned. In this diverse ensemble, XGBoost emerges as a standout performer, marked by its exceptional accuracy and recall scores, coupled with a harmoniously balanced F1 Score and precision on the Cardiovascular Heart Disease Dataset. This points out XGBoost’s transformative potential in the realm of heart disease prediction and diagnosis, positioning it as an invaluable tool for healthcare professionals. The model instills a high level of confidence in identifying potential cases of heart disease, firmly establishing itself as an exemplary choice within this dataset. The exceptional precision and accuracy exhibited by these models bear profound implications for the diagnosis and care of individuals with heart disease. Such precision not only enhances diagnostic accuracy but also opens new avenues for interventions and treatments that can be initiated with heightened confidence. In the quest for the most suitable model, it is imperative to align the selection with the specific requirements and constraints of the application at hand. Practical considerations such as interpretability, computational complexity, and data availability should guide the decision-making process, ensuring that the chosen model is tailored to meet the unique needs of the task. These findings culminate in a valuable resource that can empower informed decision-making within the realm of heart disease prediction, particularly in clinical settings. The potential to revolutionize heart disease diagnosis and patient care is emphasized, further cementing the significance of machine learning in the field of healthcare. In practical terms, this implies that when the model indicates an individual as having heart disease, the likelihood of accuracy is notably high, signifying a significant advancement in the landscape of medical diagnostics. Future directions for this study could involve expanding the scope by incorporating more extensive medical imaging datasets. Leveraging such data could enhance image-based heart disease prediction, potentially leading to even more accurate and robust diagnostic tools in the field of cardiovascular health. Furthermore, exploring ensemble models that merge the strengths of multiple algorithms may offer promising avenues for further improving predictive accuracy in the field of heart disease prediction. These considerations shed light on the multifaceted nature of heart disease prediction research, emphasizing the need for ongoing refinement and innovation in this critical domain. Future research directions should also prioritize the refinement of models and expansion of datasets. In contrast to [42,43], our study employs a distinct dataset, leveraging its unique characteristics to enhance the robustness and generalizability of the models. Furthermore, the selection of machine learning models in our work deviates from those used in the cited studies, contributing to the innovative aspect of our approach. Importantly, the outcomes of our models exhibit a noteworthy improvement in predictive accuracy, establishing a superior performance benchmark.

This nuanced combination of dataset, model selection, and elevated accuracy underscores the distinctive contribution of our work to the field of heart disease prediction. It positions our study as an advancement beyond existing research, offering a more refined and accurate predictive framework.

## Figures and Tables

**Table 1:** Summary of the performance of the existing methods.

Study	Method	Results
Mohan et al. [21]	Hybrid Random Forest with Linear Model (HRFLM)	Accuracy: 88% Sensitivity: 92.8% Specificity: 82.6%
Singh et al. [22]	SVM K-Nearest Neighbors Decision Tree Linear Regression	83% Accuracy SVM 79% (DT) 78% (LR) 87% (KNN)
Gavhane et al. [23]	Neural Network	Precision rate: 91% Recall rate: 89%
Kavitha et al. [24]	Hybrid Model (Random Forest (RF) and Decision Tree (DT))	Accuracy: 88%
Amiri and Armano [25]	Classification—CART	Accuracy: 99.14% Sensitivity: 100% Specificity: 98.28%
Liu and Kim [26]	Classifier—Long Short Term Memory (LSTM)	Accuracy: 98.4%

**Table 2:** Preprocessing and predictive methods.

Study	Dataset	Preprocessing and Modeling	Results
Algarni et al. [27]	Coronary artery X-ray angiography images obtained from a clinical database.	Training: 100 images Test: 30 images ASCARIS model (based on color, diameter, and shape features).	Accuracy: 97%
Uyar and İlhan [30]	Cleveland dataset for heart disease.	Removal of 6 instances with missing entries from the dataset and categorization of the diagnosis attribute (num) into two classes: absence (num = 0) and presence (num = 1, 2, 3, or 4) of heart disease. Recursive Fuzzy Neural Network (RFNN)	Testing set accuracy: 97.78% Overall accuracy: 96.63%
Deng et al. [31]	Fuwai ECG database and public PTB database	training phase for dynamics acquisition and a test phase for dynamics reuse Attention-based Res-BiLSTM-Net model	F1 scores ranging from 0.72 to 0.98
Das et al. [32]	UCI dataset	SAS-based software Neural Networks	Training accuracy: 86.4%, Validation accuracy: 89.011%

**Table 3:** Cardiovascular Heart Disease Dataset.

Features	Details
1. Patient Id	Individual unique identifier.
2. Age	Numeric representation of patients’ age in years.
3. Gender	Binary (1, 0 (0 = female, 1 = male))
4. Chestpain	Nominal (0, 1, 2, 3 (Value 0: typical angina Value 1: atypical angina Value 2: non-anginal pain Value 3: asymptomatic))
5. restingBP	Numeric (94–200 (in mm HG))
6. serumcholestrol	Numeric (126–564 (in mg/dL))
7. fastingbloodsugar	Binary (0, 1 > 120 mg/dL (0 = false, 1 = true))
8. restingrelectro	Nominal (0, 1, 2 (Value 0: normal, Value 1: having ST-T wave abnormality (T wave inversions and/or ST elevation or depression of >0.05 mV), Value 2: showing probable or definite left ventricular hypertrophy by Estes’ criteria))
9. maxheartrate	Numeric (71–202)
10. exerciseangia	Binary (0, 1 (0 = no, 1 = yes))
11. oldpeak	Numeric (0–6.2)
12. slope	Nominal (1, 2, 3 (1-upsloping, 2-flat, 3-downsloping))
13. noofmajorvessels	Numeric (0, 1, 2, 3)
14. target	Binary (0, 1 (0 = Absence of Heart Disease, 1= Presence of Heart Disease))

**Table 4:** Heart Disease Cleveland Dataset.

Features	Details
1. Age	Numeric representation of patients’ age in years.
2. Sex	Categorical feature representing gender, where Male is encoded as 1 and Female as 0.
3. cp	Categorical attribute indicating the various types of chest pain felt by the patient. 0 for typical angina, 1 for atypical angina, 2 for non-anginal pain, and 3 for asymptomatic.
4. trestbps	Numerical measurement of the patient’s blood pressure at rest, recorded in mm/HG.
5. chol	Numeric value indicating the serum cholesterol intensity of the patient, calculated in mg/dL.
6. fbs	Categorical representation of fasting blood sugar levels, with 1 signifying levels above 120 mg/dL and 0 indicating levels below.
7. restecg	Categorical feature describing the result of the electrocardiogram conducted at rest. 0 for normal, 1 for ST-T wave abnormalities, and 2 for indications of probable or definite left ventricular hypertrophy according to Estes’ criteria.
8. thalach	Numeric representation of the heart rate realized by the patient.
9. exang	Categorical feature denoting whether exercise-induced angina is present. 0 signifies no, while 1 signifies yes.
10. oldpeak	Numeric value indicating exercise-induced ST-depression relative to the rest state.
11. slope	Categorical attribute representing the slope of the ST segment during peak exercise. It can take three values: 0 for up-sloping, 1 for flat, and 2 for down-sloping.
12. ca	Categorical feature indicating the number of major blood vessels, ranging from 0 to 3.
13 thal	Categorical representation of a blood disorder called thalassemia. 0 for NULL, 1 for normal blood flow, 2 for fixed defects (indicating no blood flow in a portion of the heart), and 3 for reversible defects (indicating abnormal but observable blood flow).
14. target	The target variable to predict heart disease, encoded as 1 for patients with heart disease and 0 for patients without heart disease.

**Table 5:** Results on Precision measure.

Classification Model	Precision (in %)	
	**Dataset 1**	**Dataset 2**
KNN	96.50%	**96.55%**
RF	98.63%	94.44%
LR	96.55%	93.10%
GB	99.13%	90.00%
SVM	95.00%	80.65%
CNN	99.14%	87.50%
XGBoost	**99.14%**	90.00%

**Table 6:** Results on Recall measure.

Classification Model	Recall (in %)	
	**Dataset 1**	**Dataset 2**
KNN	97.44%	87.50%
RF	**98.97%**	85.61%
LR	95.73%	84.38%
GB	97.44%	84.38%
SVM	97.44%	78.12%
CNN	98.29%	**89.77%**
XGBoost	98.29%	84.38%

**Table 7:** Results on F1-Score measure.

Classification Model	F1-Score (in %)	
	**Dataset 1**	**Dataset 2**
KNN	97.02%	**91.80%**
RF	**98.80%**	89.81%
LR	96.14%	88.52%
GB	98.28%	87.10%
SVM	96.20%	79.37%
CNN	97.80%	87.50%
XGBoost	98.71%	87.10%

**Table 8:** Results on Accuracy measure.

Classification Model	Accuracy (in %)	
	**Dataset 1**	**Dataset 2**
KNN	96.50%	**91.80%**
RF	**98.60%**	91.09%
LR	95.50%	88.52%
GB	98.00%	86.89%
SVM	95.50%	78.69%
CNN	97.50%	86.89%
XGBoost	98.50%	86.89%

## Data Availability

The datasets are available online and upon request.
